# Supplementary material for: Resolving the full spectrum of human genome variation using Linked-Reads
Source: Genome Res. 2019 Apr;29(4):635–45. doi: 10.1101/gr.234443.118 (PMC6442396; doi:10.1101/gr.234443.118)
Supplement: Supplemental Material [file supp_gr.234443.118_Supplemental_longranger-2.2.zip › longranger-2.2.tar.bz2!/lr2.2-public/lib/stan/cmdstan/stan/doc/api/html/agrad_2rev_2matrix_2trace__quad__form_8hpp_source.html]

Stan: src/stan/agrad/rev/matrix/trace\_quad\_form.hpp Source File


|  |  |
| --- | --- |
|  | Stan  2.5.0  probability, sampling & optimization |


- Main Page
- Related Pages
- Namespaces
- Classes
- Files

- File List
- File Members

All Classes Namespaces Files Functions Variables Typedefs Enumerations Enumerator Friends Macros Pages

- src
- stan
- agrad
- rev
- matrix

trace\_quad\_form.hpp

Go to the documentation of this file.

1 #ifndef STAN\_\_AGRAD\_\_REV\_\_MATRIX\_\_TRACE\_QUAD\_FORM\_HPP

2 #define STAN\_\_AGRAD\_\_REV\_\_MATRIX\_\_TRACE\_QUAD\_FORM\_HPP

3

4 #include <boost/utility/enable\_if.hpp>

5 #include <boost/type\_traits.hpp>

6 #include <stan/math/matrix/Eigen.hpp>

7 #include <stan/math/matrix/typedefs.hpp>

8 #include <stan/agrad/rev/matrix/value\_of.hpp>

9 #include <stan/agrad/rev/var.hpp>

10 #include <stan/agrad/rev/vari.hpp>

11 #include <stan/agrad/rev/matrix/typedefs.hpp>

12 #include <stan/math/matrix/trace\_quad\_form.hpp>

13 #include <stan/math/error\_handling/matrix/check\_multiplicable.hpp>

14 #include <stan/math/error\_handling/matrix/check\_square.hpp>

15

16 namespace stan {

17  namespace agrad {

18  namespace {

19  template<typename TA,int RA,int CA,typename TB,int RB,int CB>

20  class trace\_quad\_form\_vari\_alloc : public chainable\_alloc {

21  public:

22  trace\_quad\_form\_vari\_alloc(const Eigen::Matrix<TA,RA,CA> &A,

23  const Eigen::Matrix<TB,RB,CB> &B)

24  : A\_(A), B\_(B)

25  { }

26

27  double compute() {

28  return stan::math::trace\_quad\_form(value\_of(A\_),

29  value\_of(B\_));

30  }

31

32  Eigen::Matrix<TA,RA,CA> A\_;

33  Eigen::Matrix<TB,RB,CB> B\_;

34  };

35

36  template<typename TA,int RA,int CA,typename TB,int RB,int CB>

37  class trace\_quad\_form\_vari : public vari {

38  protected:

39  static inline void chainA(Eigen::Matrix<double,RA,CA> &A,

40  const Eigen::Matrix<double,RB,CB> &Bd,

41  const double &adjC) {}

42  static inline void chainB(Eigen::Matrix<double,RB,CB> &B,

43  const Eigen::Matrix<double,RA,CA> &Ad,

44  const Eigen::Matrix<double,RB,CB> &Bd,

45  const double &adjC) {}

46

47  static inline void chainA(Eigen::Matrix<var,RA,CA> &A,

48  const Eigen::Matrix<double,RB,CB> &Bd,

49  const double &adjC)

50  {

51  int i,j;

52  Eigen::Matrix<double,RA,CA> adjA(adjC\*Bd\*Bd.transpose());

53  for (j = 0; j < A.cols(); j++)

54  for (i = 0; i < A.rows(); i++)

55  A(i,j).vi\_->adj\_ += adjA(i,j);

56  }

57  static inline void chainB(Eigen::Matrix<var,RB,CB> &B,

58  const Eigen::Matrix<double,RA,CA> &Ad,

59  const Eigen::Matrix<double,RB,CB> &Bd,

60  const double &adjC)

61  {

62  int i,j;

63  Eigen::Matrix<double,RA,CA> adjB(adjC\*(Ad + Ad.transpose())\*Bd);

64  for (j = 0; j < B.cols(); j++)

65  for (i = 0; i < B.rows(); i++)

66  B(i,j).vi\_->adj\_ += adjB(i,j);

67  }

68

69  inline void chainAB(Eigen::Matrix<TA,RA,CA> &A,

70  Eigen::Matrix<TB,RB,CB> &B,

71  const Eigen::Matrix<double,RA,CA> &Ad,

72  const Eigen::Matrix<double,RB,CB> &Bd,

73  const double &adjC)

74  {

75  chainA(A,Bd,adjC);

76  chainB(B,Ad,Bd,adjC);

77  }

78

79

80  public:

81  trace\_quad\_form\_vari(trace\_quad\_form\_vari\_alloc<TA,RA,CA,TB,RB,CB> \*impl)

82  : vari(impl->compute()), \_impl(impl) { }

83

84  virtual void chain() {

85  chainAB(\_impl->A\_, \_impl->B\_,

86  value\_of(\_impl->A\_), value\_of(\_impl->B\_),

87  adj\_);

88  };

89

90  trace\_quad\_form\_vari\_alloc<TA,RA,CA,TB,RB,CB> \*\_impl;

91  };

92  }

93

94  template<typename TA,int RA,int CA,typename TB,int RB,int CB>

95  inline typename

96  boost::enable\_if\_c< boost::is\_same<TA,var>::value ||

97  boost::is\_same<TB,var>::value,

98  var >::type

99  trace\_quad\_form(const Eigen::Matrix<TA,RA,CA> &A,

100  const Eigen::Matrix<TB,RB,CB> &B)

101  {

102  stan::math::check\_square("trace\_quad\_form(%1%)",A,"A",(double\*)0);

103  stan::math::check\_multiplicable("trace\_quad\_form(%1%)",A,"A",

104  B,"B",(double\*)0);

105

106  trace\_quad\_form\_vari\_alloc<TA,RA,CA,TB,RB,CB> \*baseVari = new trace\_quad\_form\_vari\_alloc<TA,RA,CA,TB,RB,CB>(A,B);

107

108  return var(new trace\_quad\_form\_vari<TA,RA,CA,TB,RB,CB>(baseVari));

109  }

110  }

111 }

112

113 #endif

check\_multiplicable.hpp

vari.hpp

typedefs.hpp

typedefs.hpp

trace\_quad\_form.hpp

stan::agrad::value\_of

T value\_of(const fvar< T > &v)

Return the value of the specified variable.

**Definition:** value\_of.hpp:16

B\_

Eigen::Matrix< TB, RB, CB > B\_

**Definition:** trace\_quad\_form.hpp:33

stan::agrad::trace\_quad\_form

stan::agrad::fvar< T > trace\_quad\_form(const Eigen::Matrix< stan::agrad::fvar< T >, RA, CA > &A, const Eigen::Matrix< stan::agrad::fvar< T >, RB, CB > &B)

**Definition:** trace\_quad\_form.hpp:19

stan::math::check\_multiplicable

bool check\_multiplicable(const char \*function, const T1 &y1, const char \*name1, const T2 &y2, const char \*name2, T\_result \*result)

**Definition:** check\_multiplicable.hpp:15

value\_of.hpp

stan::math::trace\_quad\_form

double trace\_quad\_form(const Eigen::Matrix< double, RA, CA > &A, const Eigen::Matrix< double, RB, CB > &B)

Compute trace(B^T A B).

**Definition:** trace\_quad\_form.hpp:17

\_impl

trace\_quad\_form\_vari\_alloc< TA, RA, CA, TB, RB, CB > \* \_impl

**Definition:** trace\_quad\_form.hpp:88

var.hpp

Eigen.hpp

stan::agrad::var

Independent (input) and dependent (output) variables for gradients.

**Definition:** var.hpp:27

stan::math::check\_square

bool check\_square(const char \*function, const Eigen::Matrix< T\_y, Eigen::Dynamic, Eigen::Dynamic > &y, const char \*name, T\_result \*result)

Return true if the specified matrix is square.

**Definition:** check\_square.hpp:24

A\_

Eigen::Matrix< TA, RA, CA > A\_

**Definition:** trace\_quad\_form.hpp:32

check\_square.hpp


---

[ Stan Home Page ]

*© 2011–2014,
Stan Development Team.*
